# Supplementary material for: Electronic Skin Based on Polydopamine-Modified Superelastic Fibers with Superior Conductivity and Durability
Source: Nanomaterials (Basel). 2024 Feb 28;14(5):438. doi: 10.3390/nano14050438 (PMC10934599; doi:10.3390/nano14050438)
Supplement: Supplementary file 1 [file nanomaterials-14-00438-s001.zip › nanomaterials-2859460-supplementary.pdf]

Supplementary Materials for

Electronic Skin Based on Polydopamine-Modified Superelastic Fibers with Superior Conductivity and Durability

Chengfeng Chen [1,2], Yimiao Wang [1], Hang Wang [1,2,*], Xinqing Wang [1] and Mingwei Tian [1,*]

[1] Intelligent Wearable Engineering Research Center of Qingdao, College of Textiles and Clothing, Qingdao University, Qingdao 266071, China
[2] Shandong Special Nonwoven Materials Engineering Research Center, Qingdao University, Qingdao 266071, China
* Correspondence: wanghang@qdu.edu.cn (H.W.); mwtian@qdu.edu.cn (M.T.)

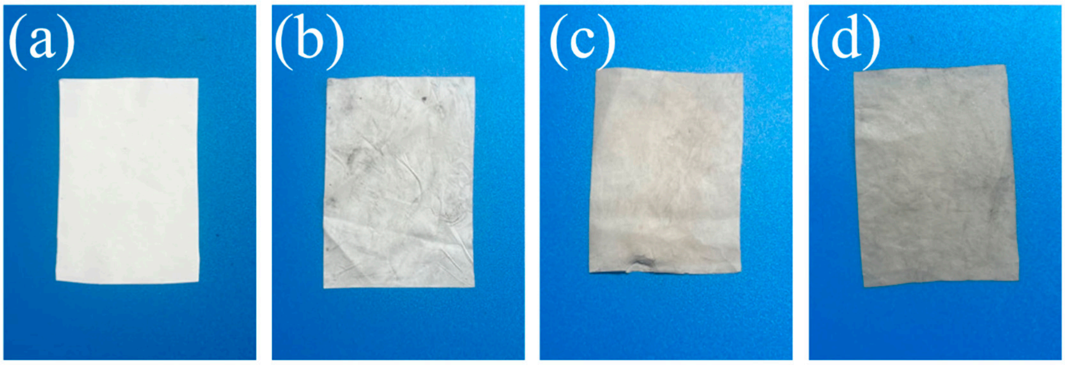

Supporting Figure S1 (a–d) PF, DPF-3, DPF-6, DPF-9 fibre mesh pictures.

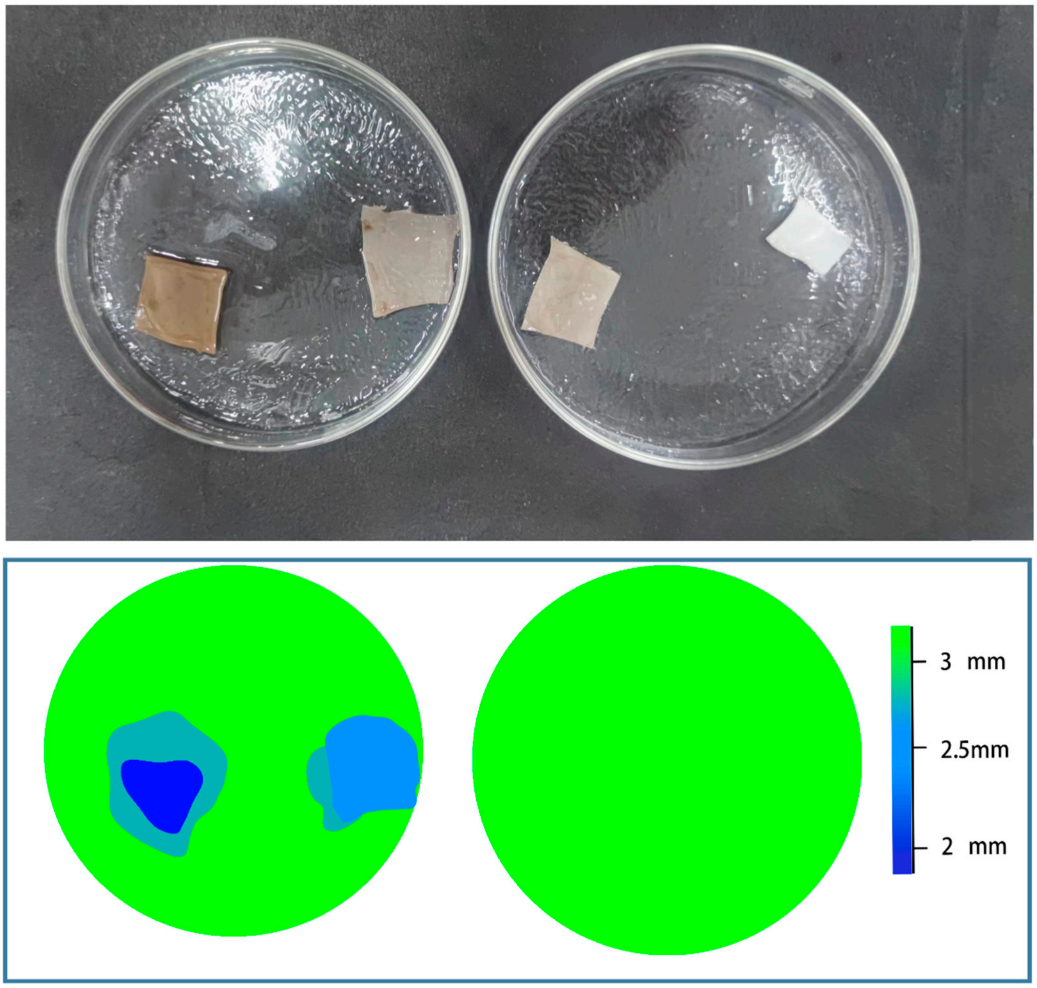

Supporting Figure S2. Solidification of different fibre networks in hydrogels.

The various fiber mesh specimens underwent a 40-minute heat treatment at 90°C within the hydrogel precursor. The solidification of the hydrogel around these distinct fiber samples was scrutinized. The hydrogel encasing the PF and DPF-3 samples solidified entirely, forming a 3 mm thickness. In contrast, portions of the hydrogel surrounding the DPF-6 and DPF-9 samples did not achieve full solidification.

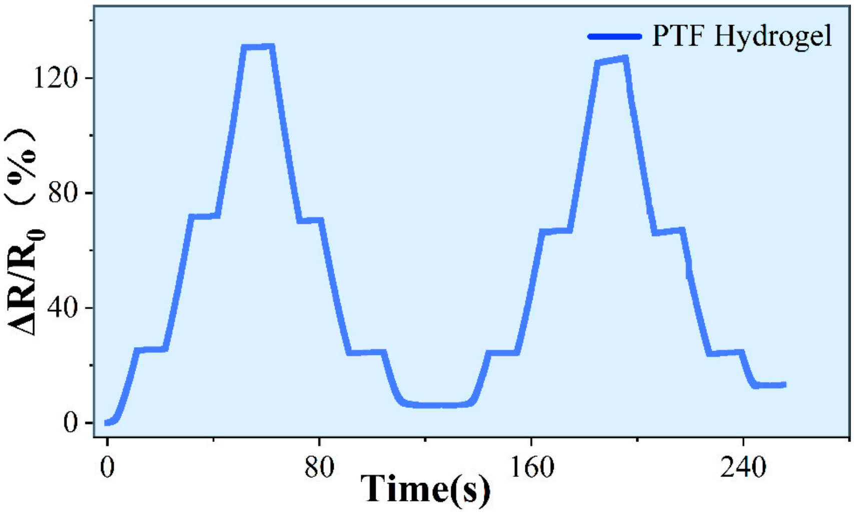

Supporting Figure S3. The DPF hydrogel exhibits electrical signal variations during the stretching-resting process.

Electrical signal fluctuations of the sensor were obtained throughout the motion–stationary drafting process at 10 s intervals on a drafting machine. The simulation emulates human joint movements to assess the stability of the sensor. The sensor exhibited diverse changes correlating with motion, signifying its resistance to drifting and showcasing its substantial potential for motion detection.

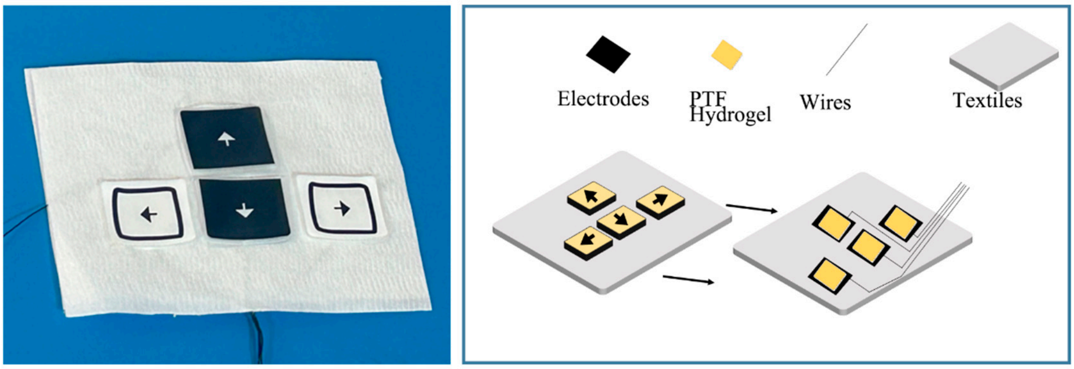

Supporting Figure S4. Flexible keyboard and its structural composition crafted from DPF hydrogel.

The flexible keyboard comprises hydrogel, electrodes, wiring, fabric, and an external circuit board. The hydrogel functions as a resistive sensor within the flexible keyboard, discerning touch or key press actions and transforming them into corresponding electrical signals.
